# Toll-Like Receptor (*TLR*) and Nucleosome-binding Oligomerization Domain (*NOD*) gene polymorphisms and endometrial cancer risk

**DOI:** 10.1186/1471-2407-10-382

**Published:** 2010-07-21

**Authors:** Katie A Ashton, Anthony Proietto, Geoffrey Otton, Ian Symonds, Mark McEvoy, John Attia, Rodney J Scott

**Affiliations:** 1 Discipline of Medical Genetics, School of Biomedical Sciences, Faculty of Health, University of Newcastle, Australia and the Hunter Medical Research Institute, NSW, 2305, Australia; 2 Hunter Centre for Gynaecological Cancer, John Hunter Hospital, Newcastle, NSW, 2305, Australia; 3 School of Medicine and Public Health, Faculty of Health, University of Newcastle, NSW, 2305, Australia; 4 Division of Genetics, Hunter Area Pathology Service, John Hunter Hospital, Newcastle, NSW, 2305, Australia

## Abstract

**Background:**

Endometrial cancer is the most common gynaecological malignancy in women of developed countries. Many risk factors implicated in endometrial cancer trigger inflammatory events; therefore, alterations in immune response may predispose an individual to disease. Toll-like receptors (*TLRs*) and nucleosome-binding oligomerization domain (*NOD*) genes are integral to the recognition of pathogens and are highly polymorphic. For these reasons, the aim of the study was to assess the frequency of polymorphic variants in *TLR *and *NOD *genes in an Australian endometrial cancer population.

**Methods:**

Ten polymorphisms were genotyped in 191 endometrial cancer cases and 291 controls using real-time PCR: *NOD1 *(rs2075822, rs2907749, rs2907748), *NOD2 *(rs5743260, rs2066844, rs2066845), *TLR2 *(rs5743708), *TLR4 *(rs4986790) and *TLR9 *(rs5743836, rs187084).

**Results:**

Haplotype analysis revealed that the combination of the variant alleles of the two *TLR9 *polymorphisms, rs5743836 and rs187084, were protective for endometrial cancer risk: OR 0.11, 95% CI (0.03-0.44), p = 0.002. This result remained highly significant after adjustment for endometrial cancer risk factors and Bonferroni correction for multiple testing. There were no other associations observed for the other polymorphisms in *TLR2*, *TLR4*, *NOD1 *and *NOD2*.

**Conclusions:**

The variant 'C' allele of rs5743836 causes greater *TLR9 *transcriptional activity compared to the 'T' allele, therefore, higher *TLR9 *activity may be related to efficient removal of microbial pathogens within the endometrium. Clearly, the association of these *TLR9 *polymorphisms and endometrial cancer risk must be further examined in an independent population. The results point towards the importance of examining immune response in endometrial tumourigenesis to understand new pathways that may be implicated in disease.

## Background

Endometrial cancer represents a significant burden on society as it is the most common gynaecological malignancy diagnosed in women of developed countries [1-3]. Extensive analysis of the reproductive and environmental factors implicated in disease has been undertaken, revealing that excessive/unopposed estrogen is the major risk factor for disease; however, the genetic events underlying the development and progression of endometrial cancer are not well understood [4]. In 2005, Modugno *et al*. proposed that inflammation may play a role in the development of endometrial cancer [5]. This hypothesis was based on the observation that the menstrual cycle resembles an inflammatory process and that risk factors associated with endometrial cancer such as early menstruation, late menopause and exposure to unopposed estrogens, may increase exposure to inflammatory events [5]. Support for altered immune response in endometrial cancer is also evident in a number of other related diseases such as endometrial hyperplasia, a precursor to endometrial cancer development, and in endometriosis, a common benign gynaecological condition [5-7]. Many studies have shown an association between inflammation and cancer [8], therefore, individual differences in response to inflammation may alter the risk of developing endometrial cancer.

The maintenance of the immune response is important for the recognition and subsequent response to a wide range of microbial pathogens [9]. To determine differences between the pathogen associated molecular patterns (PAMPs) of these microbes, the innate immune system uses specific pattern recognition receptors (PRRs) to elicit an immune response [9]. Two specific examples of PRRs are: toll-like receptors (TLRs) and intracellular nucleosome-binding oligomerisation domain (NOD) proteins.

The TLR family consists of 10 membrane-bound proteins that respond to microbes in the extracellular matrix [10] whereas the NOD proteins, NOD1 and NOD2, recognise microbial pathogens located in the cytoplasm [11]. Polymorphic variants in these genes have been studied in a number of inflammatory diseases and cancer however their association with endometrial cancer remains elusive. With respect to the *TLR*s, *TLR2*, *TLR4 *and *TLR9 *are highly polymorphic and represent interesting targets to elucidate their role in endometrial cancer development [12]. *TLR2 *recognises PAMPs from a wide range of pathogens; *TLR4 *is involved in the recognition of bacterial lipopolysaccharide (LPS) and *TLR9 *recognises unmethylated CpG motifs present in bacterial DNA and intracellular antigens [10,13,14]. Furthermore, *NOD1 *and *NOD2 *are involved in the recognition of many bacterial pathogens and their activation signals the transcription of many pro-inflammatory genes following NF-κB activation [11]. Genetic variants in *NOD1 *and *NOD2*, have been associated with Crohn's disease and other inflammatory disorders [11].

The aim of this study was to examine 10 polymorphisms in *NOD1 *(rs2075822, rs2907749 and rs2907748), *NOD2 *(rs5743260, rs2066844 and rs2066845), *TLR2 *(rs5743708), *TLR4 *(rs4986790) and *TLR9 *(rs5743836 and rs187084) to determine whether there was any association with endometrial cancer risk in an age and sex matched Caucasian population.

## Methods

### Study Population

The study population has been previously described [15-17]. This study initially consisted of 213 consecutively recruited women with histologically confirmed endometrial cancer who presented for treatment at the Hunter Centre for Gynaecological Cancer, John Hunter Hospital, Newcastle, New South Wales, Australia between the years 1992 and 2005. Women that had additionally been diagnosed with breast cancer were excluded from this study.

The final analysis included 191 endometrial cancer patients. Data on reproductive and environmental risk factors including ethnicity, body mass index (BMI), diabetes, high blood pressure (HBP), age of diagnosis of endometrial cancer, age of menarche, age of menopause, other personal cancer history, family cancer history (Family history of cancer was defined as cancer in the index patient plus one or more 1st or 2nd degree relatives diagnosed with cancer), parity, breastfeeding, oral contraceptive use, chemotherapy, radiotherapy, hormone therapy (HT), smoking and alcohol use was collected using self reported questionnaires. Information regarding recurrence, stage, grade and histology of endometrial cancer was collected from their medical records.

The control population consisted of 291 women that were recruited between the years 2004 and 2005 for the Hunter Community Study. This study aims to identify genetic and environmental factors associated with ageing in a cohort of individuals obtained from the Hunter region, Newcastle, New South Wales, Australia. Any control that had a prior diagnosis of either breast or endometrial cancer was excluded from the study. Controls were matched to cases by sex and age.

All participants provided informed written consent prior to participation in this study. Ethics approval was obtained from the Human Research Ethics Committee, University of Newcastle, New South Wales, Australia (H-050-0605) and the Hunter Area Research Ethics Committee, Hunter New England Health Service, Newcastle, New South Wales, Australia (05/03/09/3.14).

### DNA Isolation

Genomic DNA was extracted from 10 ml EDTA blood using the "salting-out" method [18].

### Molecular Analysis

Genotyping of three *NOD1 *polymorphisms (rs2075822, rs2907749 and rs2907748); three *NOD2 *polymorphisms (rs5743260, rs2066844 and rs2066845), one *TLR2 *polymorphism (rs5743708), one *TLR4 *polymorphism (rs4986790) and two *TLR9 *polymorphisms (rs5743836 and rs187084) was performed on an ABI PRISM^® ^7500 Real-Time PCR System (PE Applied Biosystems, Foster City, CA), using primers and probes from the Assay-by-Design service (Applied Biosystems). The primer and probe sequences are available upon request. The assay was performed under universal conditions as previously described (Ashton *et al*., 2008). The genotyping results were confirmed by a second laboratory research assistant and 5% of the samples were re-genotyped with 100% concordance. Any sample where a genotype could not be accurately assessed was re-genotyped. The overall call rates were 100%.

### Statistical Analysis

Power calculations were performed using Quanto (Version 1.2.3, May 2007, http://hydra.usc.edu/GxE). The calculations performed showed that the number of cases and controls (ratio 1:1.52) in our cohort was large enough to detect a significant, p < 0.05, 2-fold increased risk (OR >2.0), with 80% power, assuming a dominant genetic model, with a minor allele frequency of 6.5%. Therefore, our study has a large enough sample size to statistically demonstrate that significant OR values over 2.0 or below 0.5 provide a statistically robust result.

For each polymorphism, Hardy-Weinberg Equilibrium (HWE) was calculated in the control group to check for compliance using the Institute for Human Genetics, statistics website, http://ihg2.helmholtz-muenchen.de/cgi-bin/hw/hwa1.pl (Munich, Germany). To determine differences in genotype frequencies and environmental and reproductive risk factors between the cases and controls, chi-squared (χ^2^) statistics, odds ratios (ORs) and 95% confidence intervals (CI) were calculated using unconditional logistic regression. Multivariate unconditional logistic regression was performed to determine if any risk factors altered the significance of the genotype frequency results. The risk factors taken into account were: BMI (<25 kg/m^2 ^and ≥25 kg/m^2^), diabetes (yes/no), HBP (yes/no), HT (yes/no), personal history of cancer (yes/no), smoking (ever/never) and alcohol consumption (ever/never). Other risk factors such as age of menopause were not included in the analysis since this information was not available for the controls.

The genotype frequencies of all polymorphisms were compared in the case group stratified for the environmental and reproductive risk factors by using chi-squared (χ^2^) analysis and ORs and 95% CI were calculated using unconditional logistic regression. To avoid chance findings due to multiple testing of the 10 polymorphisms and 16 environmental/reproductive risk factors, Bonferroni correction was used and the significance levels was lowered to p = 0.005 (p = 0.05/10) for the 10 polymorphisms and p = 0.003 (p = 0.05/16) for the 16 environmental factors.

T-tests were used to determine differences in the age of diagnosis of endometrial cancer by genotype. Kaplan Meier survival analysis was used to plot the cumulative survival versus the patient's age of diagnosis of endometrial cancer. By comparing the Kaplan-Meier survival curves for each genotype, differences in the age of diagnosis of endometrial cancer by genotype were examined. The Wilcoxon's test was used to determine the significance of observations from early ages of diagnosis, log-rank test, which gives more weight to later ages and Tarone-Ware test, which is an intermediate of the two other tests were used to examine the homogeneity of the survival curves. The polymorphisms that showed a statistically significant difference between the genotypes and the age of diagnosis of endometrial cancer for all three statistical tests were further examined by a multivariate Cox regression model where the risk factors listed above were incorporated into the analysis.

Haplotypes were estimated using SIMHAP [19]. Linkage disequilibrium (LD) was tested applying Lewontin's D' statistic using the pwld function in STATA. Associations of single haplotypes and combinations of haplotypes with endometrial cancer risk were performed using SIMHAP.

The significance levels of all tests were set at p < 0.05 and were two-sided. All statistical analysis was performed with SIMHAP (Laboratory for Genetic Epidemiology, Western Australian Institute for Medical Research, Australia), Intercooled STATA 8.2 (Stata Corp., College Station, TX, USA) and SPSS Version 15 (SPSS Inc. Chicago, IL, USA).

## Results

### Comparison of selected environmental and reproductive risk factors between cases and controls

Cases and controls were different with respect to potential endometrial cancer risk factors, including HBP, diabetes, HT, alcohol consumption, personal history of any cancer, personal history of ovarian cancer, cervical cancer and other cancers as previously described [15-17].

### Hardy Weinberg Equilibrium (HWE) and Linkage Disequilibrium (LD)

The distributions of the genotypes of all ten polymorphisms in *NOD1*, *NOD2*, *TLR2*, *TLR4 *and *TLR9 *among the cases and controls did not deviate from HWE. The three *NOD1 *polymorphisms were in high LD (D' values; rs2075822 + rs2907749 = 0.89, rs2075822 + rs2907748 = 0.88 and rs2907749 + rs2907748 = 1.00). In addition, the *NOD2 *polymorphisms were in high LD (D' values; rs5743260 + rs2066844 = 1.00 and rs5743260 + rs2066845 = 1.00) however rs2066844 and rs2066845 were not in LD (D' = 0.2). None of the *TLR *polymorphisms were in high LD. Three *TLR *combinations were in partial LD: *TLR2 *rs5743708 + *TLR4 *rs4986790, D' = 0.61; *TLR2 *rs5743708 + *TLR9 *rs187084, D' = 0.57; and *TLR4 *rs4986790 + *TLR9 *rs187084, D' = 0.57.

### Comparison of genotype, allele and haplotype frequencies of polymorphisms among endometrial cancer cases and controls

The allele and genotype frequencies were compared between the cases and controls for the polymorphisms in *NOD1*, *NOD2*, *TLR2*, *TLR4 *and *TLR9 *(see Table 1). The only significant result was observed for *TLR9 *rs187084; allele T vs allele C: OR 0.675, 95% CI (0.478-0.954), p = 0.025. After Bonferroni correction for multiple testing, this result was no longer significant (p value must be less than 0.005). Haplotype analysis revealed that the combination of the variant alleles for *TLR9 *rs187084 and rs5743836 were associated with a significant decreased risk of endometrial cancer; TT vs CC: OR 0.11, 95% CI (0.03-0.44), p = 0.002 (see Table 2). This result remained highly significant after adjustment for endometrial cancer risk factors and Bonferroni correction for multiple testing; TT vs CC: OR 0.10, 95% CI (0.02-0.46), p = 0.003 (see Table 2).

**Table 1:** Genotype frequencies of polymorphisms in *NOD1, NOD2, TLR2, TLR4 *and *TLR9 *and endometrial cancer risk

Polymorphism	Genotypes	Cases n (%)	Controls n (%)	**χ**^**2**^	OR (95% CI) and p value
	TT	119 (62.3)	176 (60.5)		
*NOD1 *(rs2075822)	TC	62 (32.5)	105 (36.1)		1.188
	CC	10 (5.2)	10 (3.4)	p = 0.497	(0.774-1.823) p = 0.432
	TC+CC	72 (37.7)	115 (39.5)		
	AA	104 (54.5)	152 (52.2)		
*NOD1 *(rs2907749)	AG	68 (35.6)	118 (40.6)		1.156
	GG	19 (9.9)	21 (7.2)	p = 0.392	(0.759-1.761) p = 0.499
	AG+GG	87 (45.5)	139 (47.8)		
	CC	114 (59.7)	168 (57.7)		
*NOD1 *(rs2907748)	CT	64 (33.5)	110 (37.8)		1.156
	TT	13 (6.8)	13 (4.5)	p = 0.400	(0.755-1.769) p = 0.505
	CT+TT	77 (40.3)	123 (42.3)		
	CC	182 (95.3)	282 (96.9)		1.549
*NOD2 *(rs5743260)	CT	9 (4.7)	9 (3.1)	p = 0.359	(0.604-3.977) p = 0.363
	TT	0 (0.0)	0 (0.0)		
	CC	178 (93.2)	263 (90.4)		0.928
*NOD2 *(rs2066844)	CT	13 (6.8)	28 (9.6)	p = 0.278	(0.407-2.116) p = 0.859
	TT	0 (0.0)	0 (0.0)		
	GG	187 (97.9)	282 (96.9)		0.636
*NOD2 *(rs2066845)	GC	4 (2.1)	9 (3.1)	p = 0.508	(0.143-2.835) p = 0.553
	CC	0 (0.0)	0 (0.0)		
	GG	177 (92.7)	275 (94.5)		1.190
*TLR2 *(rs5743708)	GA	14 (7.3)	16 (5.5)	p = 0.416	(0.501-2.826) p = 0.694
	AA	0 (0.0)	0 (0.0)		
	AA	163 (85.3)	258 (88.7)		
*TLR4 *(rs4986790)	AG	25 (13.1)	31 (10.6)		1.098
	GG	3 (1.6)	2 (0.7)	p = 0.449	(0.573-2.101) p = 0.778
	AG+GG	28 (14.7)	33 (11.3)		
	TT	85 (44.5)	116 (39.9)		
*TLR9 *(rs5743836)	TC	79 (41.4)	128 (44.0)		0.781
	CC	27 (14.1)	47 (16.1)	p = 0.581	(0.511-1.195) p = 0.255
	TC+CC	106 (55.5)	175 (60.1)		
	TT	138 (72.25)	187 (64.3)		
*TLR9 *(rs187084)	TC	49 (25.65)	88 (30.2)		0.675
	CC	4 (2.1)	16 (5.5)	p = 0.076	(0.428-1.064) p = 0.091
	TC+CC	53 (27.75)	104 (35.7)		

NB: Χ^2 ^- Wild-type genotype versus heterozygous genotype versus homozygous variant genotype. Odds ratios for polymorphisms in *NOD1, TLR4 *and *TLR9 *were calculated as follows: Wild Type genotype compared to combination of heterozygous and homozygous variant genotypes. Odds ratios for polymorphisms in *NOD2 *and *TLR2 *were calculated as follows: Wild Type genotype compared to heterozygous genotype. All Odds Ratios were adjusted for age, BMI, diabetes, HBP, history of cancer, HT, smoking and alcohol use.

**Table 2:** Haplotype Analysis of *TLR9 *rs187084 (T > C) and rs5743836 (T > C) and endometrial cancer risk

Gene	Haplotype	Cases n (%)	Controls n (%)	OR (95% CI)	p value
***TLR9 *(rs187084 and rs5743836)**	TT	98 (51.3)	141 (48.5)	1.00 (reference)	
	CT	65 (34.0)	90 (30.9)	1.09 (0.75-1.57)_adj^_	p = 0.66
				0.99 (0.75-1.31)	p = 0.94
	TC	26 (13.6)	39 (13.4)	1.07 (0.63-1.80)_adj^_	p = 0.76
				0.92 (0.63-1.36)	p = 0.68
	CC	2 (1.1)	21 (7.2)	**0.10 (0.02-0.46)**_**adj^**_	**p = 0.003**
				**0.11 (0.03-0.44)**	**p = 0.002**

NB: Variant allele is underlined.

^ Odds Ratio adjusted for age, BMI, diabetes, HBP, history of cancer, HT, smoking and alcohol use.

### Influence of Genetic and Environmental/Reproductive Risk Factors on the Age of Diagnosis of Endometrial Cancer

Kaplan-Meier survival analysis and T-tests were used to evaluate the influence of the *NOD1*, *NOD2*, *TLR2*, *TLR4 *and *TLR9 *polymorphisms on the age of diagnosis of endometrial cancer. There was an age effect observed for the *NOD1 *rs2075822 polymorphism. Individuals with the heterozygous and variant genotypes combined had a later age of diagnosis (65 years) in comparison to those with the wild-type genotype (62 years): Log-Rank p = 0.028; Wilcoxon, p = 0.054; and Tarone-Ware, p = 0.039. However, after stratifying the results for the environmental/reproductive risk factors using Cox regression, the results showed a similar trend but were no longer statistically significant, p = 0.061.

### Genotype frequencies in the cases stratified for environmental/reproductive risk factors

This analysis focused on all of the polymorphisms in the cases stratified for known environmental/reproductive confounders. After the results were adjusted using Bonferroni correction, no significant results were observed.

## Discussion

Recent attention has been given to the role of *TLR*s and *NOD *genes in inflammatory disorders and oncogenesis [8] as these two receptor families interact with environmental stimuli and elicit intracellular responses to microbial pathogens. New insights into the role of these genes in disease have the potential to provide new avenues for treatment and to also identify individuals at risk. Further to this, many of these genes harbour polymorphic variants that alter protein functionality and over-representation of these risk alleles in a specific population may point towards novel biological events involved in disease initiation and progression. With respect to endometrial cancer, variants in genes involved in the innate immune response have not been investigated, however, a recent study suggested that a number of risk factors trigger inflammatory events and that an altered inflammatory response may predispose an individual to develop endometrial cancer [5]. For these reasons, we sought to determine if genetic variation in a number of *TLR*s and *NOD *genes (*TLR2*, *TLR4*, *TLR9*, *NOD1 *and *NOD2*) were associated with risk of developing endometrial cancer. These polymorphisms were chosen as they have been previously related to a number of inflammatory diseases.

In the current study, we showed that the variant alleles of the *TLR9 *polymorphisms, rs5743836 (C allele) and rs187084 (C allele) combined, are associated with a decreased risk of endometrial cancer. Even though the CC subgroup harboured a small number of patients (2 cases and 21 controls), the association observed remained highly significant following adjustment for endometrial cancer risk factors and Bonferroni correction for multiple testing, indicating a true effect. These results must be interpreted with caution due to the group sizes and confirmed in an independent and larger cohort. No other associations were observed for the variants in the *TLR2*, *TLR4*, *NOD1 *or *NOD2*.

*TLR9 *belongs to the family of nucleic acid recognition *TLR*s, specifically identifying DNA-containing unmethylated CpG motifs in bacteria [13]. Activation of *TLR9 *leads to an increase in NF-κB transcriptional activation, allowing maturation of dendritic cells and release of proinflammatory cytokines [20]. It has been suggested that the *TLR9 *polymorphisms, rs5743836 and rs187084, located in the promoter region, alter the functional ability of TLR9 [21]. The variant alleles of these polymorphisms can possibly modify the response to bacterial pathogens thereby varying inter-individual disease susceptibility. A recent study by Ng *et al*. (2009), showed that following either TNFα or lipopolysaccharide (LPS) stimulation, *TLR9 *activity, for the rs5743836 polymorphism, was greater for the C allele vector compared to the T allele vector using luciferase reporter constructs [22]. In addition, they observed an increase in NF-κB binding affinity of the C allele, indicating that enhanced transcription of NF-κB results in greater release and production of pro-inflammatory mediators. Contrary to the results reported herein, this group observed an association between increased incidence of premalignant gastric changes and the C allele of the rs5743836 polymorphism, however, we have shown that the high activity C allele of rs5743836 in combination with the C allele of rs187084, appears to be protective for endometrial cancer development. To date, there is no functional data available for the rs187084 polymorphism but since it is also located in the promoter region; it could alter the function of the promoter. Clearly, functional analysis of the *TLR9 *rs187084 polymorphism needs to be performed.

Under basal conditions, another group reported that the T allele of *TLR9 *rs5743836 polymorphism was transcribed more efficiently than the C allele, indicating greater activity of the T allele rather than the C allele [21], but as pointed out by Ng *et al*. (2009), assessing basal levels may not be appropriate when studying a disease that may be influenced by exposure to bacterial pathogens. The results of our study are however similar to those observed by Novak and colleagues. They showed that the T allele was associated with an increased risk of atopic eczema (AE) whereas the C allele was observed as protective for AE. Furthermore, a study by Lazarus *et al*. (2003) revealed that the C allele was associated with asthma [23]. Clearly, there are large differences in allelic frequency of these polymorphisms in a wide range of diseases and for this reason, further studies are required to confirm these previous results and the results presented herein.

## Conclusions

The combination of the variant C alleles of *TLR9 *rs5743836 and rs187084 appears to be protective for endometrial cancer and that the high activity C allele of rs5743836 may allow a better response to pathogenic microbes that present in the endometrium. This statement must be interpreted with caution as the biological mechanisms underlying altered immune response in individuals harbouring these polymorphic variants must be further interrogated. The results presented in this study highlight that alterations in immune response genes may vary an individual's susceptibility to develop endometrial cancer. Further studies examining genes involved in the immune response pathway are warranted and have the potential to identify new biological mechanisms involved in endometrial cancer.

## Competing interests

The authors declare that they have no competing interests.

## Authors' contributions

KAA designed and executed the study. KAA provided significant insight into the interpretation of the data and wrote the manuscript. AP and GO supplied case samples and clinical information. MM supplied control samples and clinical information. IS contributed to the design of the study. JA supplied control samples and clinical information, and assisted with statistical analysis. RJS designed the study, provided the concept and corrected the manuscript. All authors have read and approved the final version of the manuscript.

## Pre-publication history

The pre-publication history for this paper can be accessed here:

http://www.biomedcentral.com/1471-2407/10/382/prepub
